# Surgical treatment of urachal adenocarcinoma with lung metastasis: A case report and literature review

**DOI:** 10.1111/1759-7714.15481

**Published:** 2024-10-27

**Authors:** Yan Tian, Chao Ren, Lin Shi, Zhanlin Guo

**Affiliations:** ^1^ Hyperbaric Oxygen Therapy Center The Affiliated Hospital of Inner Mongolia Medical University Hohhot China; ^2^ Department of Urology Surgery Affiliated Hospital of Inner Mongolia Medical University Hohhot China; ^3^ Department of Pathology Affiliated Hospital of Inner Mongolia Medical University Hohhot China; ^4^ Department of Pathology, School of basic medicine Inner Mongolia Medical University Hohhot China; ^5^ Department of Thoracic Surgery Affiliated Hospital of Inner Mongolia Medical University Hohhot China

**Keywords:** case report, pulmonary metastasis, urachal adenocarcinoma

## Abstract

Arising from the urachal epithelial lining, the urachal carcinoma is a rare tumor, which accounts for 0.35%–0.7% of all bladder cancers. Urachal carcinoma has a higher predilection in men with median age around 50–60 years old. The most common clinical symptom is intermittent painless gross hematuria, and less‐reported presentations include suprapubic mass, dysuria, lower abdominal pain, and frequent urination. The pathological study reveals that most cases (90%) are categorized as an intestinal adenocarcinoma subtype, while other morphological variants, including mucinous, enteric, signet ring cell subtype, not otherwise specified (NOS), squamous cell carcinoma, urothelial carcinoma, sarcoma, small cell carcinoma, and undifferentiated carcinoma, totally account for about 10%. The urachal carcinoma occurs mostly in the lower segment of urachal tube and bladder dome or anterior wall. However, due to the classically silent nature of the early lesions and high malignancy, urachal carcinoma patients are commonly diagnosed in advanced stage. Treatment modalities for local recurrence or metastatic urachal cancer include surgery and chemotherapy (cisplatin and 5‐FU based‐chemotherapy). Meanwhile, the EGFR‐, PD‐L1‐, and MEK‐targeted therapies in the metastatic urachal carcinoma cases showed satisfactory response. We presented a rare case of Sheldon stage IVB urachal adenocarcinoma with pulmonary metastasis, and the patient had no progression of disease 6 months following surgical treament without chemoradiotherapy.

## Graphical abstract

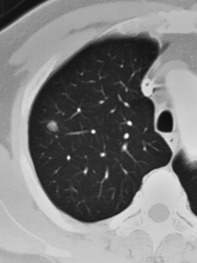

## BACKGROUND

The urachus is an embryonic remnant structure that once connected the fetal bladder to the umbilicus during development. Between the 4th and 5th months of gestation, the urachus begins to involute, eventually forming the median umbilical ligament. It lies in the loose connective tissue between the fascia transversalis and the peritoneum (known as Retropubic space). Failure of the closure process leads to various abnormalities, including malignancies due to the abnormal cell proliferation.^1^ The urachal wall consists of three layers: urothelium, connective tissue, and resident smooth muscle. Urachal carcinoma, a rare tumor arising from the epithelial lining of the urachus, accounts for only 0.35% to 0.7% of all bladder cancers.^2^ The underlying mechanism of urachal carcinoma remains unclear, but some studies suggested the involvement of urothelial intestinal metaplasia.^3^ The urachal carcinoma tends to affect men more often, with a median age of diagnosis between 50 and 60 years. The most common clinical symptom is intermittent painless gross hematuria. Less common symptoms include suprapubic masses, dysuria, lower abdominal pain, and increased urinary frequency. Pathologically, most urachal carcinomas (90%) are classified as intestinal adenocarcinomas, with the remaining 10% comprising other variants, including mucinous, enteric, signet ring cell subtype, not otherwise specified (NOS), squamous cell carcinoma, urothelial carcinoma, sarcoma, small cell carcinoma, and undifferentiated carcinoma.^4^ Urachal carcinoma typically arises in the lower urachal tube, bladder dome, or anterior wall. However, due to its often‐silent early stages and aggressive nature, it is frequently diagnosed at an advanced stage. A large retrospective study reported a median overall survival of 48 months, with a 5‐year survival rate of 45%.^5^ In metastatic cases, the median survival ranges from 12 to 24 months.^6^ The well accepted two staging systems for urachal carcinoma were proposed by Sheldon et al.^7^ and Mayo Clinic,^8^ separately. Recent studies have shown that urachal and colorectal carcinomas share similar histopathological and molecular features, including microsatellite instability (MSH2, MSH6), RAS mutations, and BRAF mutations.^9^ Treatment options for local recurrence or metastatic disease include surgery and cisplatin‐ or 5‐FU‐based chemotherapy.^1^ Additionally, targeted therapies, such as EGFR, PD‐L1, and MEK inhibitors, have shown promising results in metastatic cases.^9^, ^10^ Here, we present a rare case of urachal adenocarcinoma of IVB stage (Sheldon‐staging system) with pulmonary metastasis, And the patient had no disease progression at 6 months post‐surgery without further chemoradiotherapy.

## CASE PRESENTATION

**FIGURE 1:**
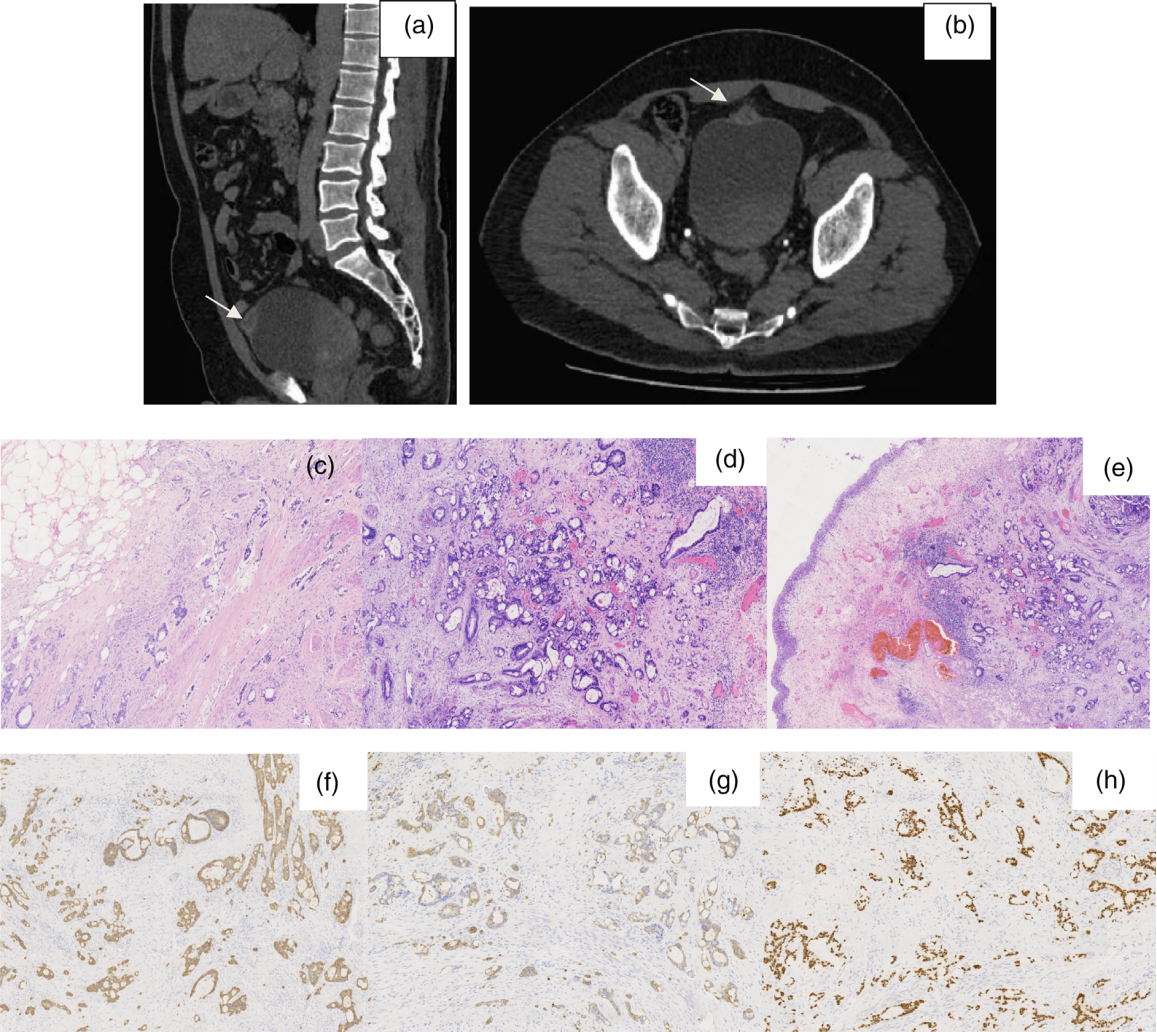
CT images of the abdomen and pelvis revealed a solid mass in the anterior wall of bladder. (a) Sagittal plane. (b) Transverse plane. (c) The tumor penetrated the bladder muscularis propria into adipose tissue; (d) Histopathological evaluation of bladder mass revealed an invasive adenocarcinoma, moderately differentiated, mucinous subtype with small to medium ducts formation, producing abundant extracellular mucin; (e) The tumor did not reach to the mucosal surface of bladder. (f) The bladder mass exhibited positive expression for CK20; (g) The bladder mass exhibited positive expression for CK7; (h) The bladder mass exhibited positive expression for CDX‐2.

A 48‐year‐old male presented with intermittent painless gross hematuria lasting over 20 days. He was hospitalized in September 2017 for further evaluation. Urinary ultrasonography revealed a hypo‐echoic nodule on the anterior bladder wall (1.7 × 1.1 cm in size) with unclear boundaries. Cystoscopy revealed a broad‐based, non‐papillary tumor in the same location. Enhanced computed tomography (CT) of the urinary system showed a solid mass of the same size in the bladder's anterior wall (Figure 1a,b).

During surgery, a sessile mass (2.5 × 2 cm in size) was found in the anterior bladder wall, sharply demarcated from the surrounding normal mucosa. A mass resection, including part of the adjacent normal mucosa, was performed under cystoscopy. Histopathological analysis from intraoperative consultation suggested an adenocarcinoma of urachal origin. Extending partial cystectomy with resection of the umbilicus, urachus, and peritoneum was subsequently performed under general anesthesia. Histological examination revealed mucinous adenocarcinoma penetrating the bladder muscularis propria into the adipose tissue, while the mucosal surface was uninvolved (Figure 1c–e). Immunohistochemistry (IHC) showed positive expression of CK20, CK7, CDX‐2 (Figure 1f–h), P53, and a Ki67‐label index of 70%, with negative p63, GATA3, and high molecular cytokeratin (HCW). The final diagnosis of the tumor was moderately differentiated urachal mucinous adenocarcinoma based on imaging, histology, and IHC findings.

**FIGURE 2:**
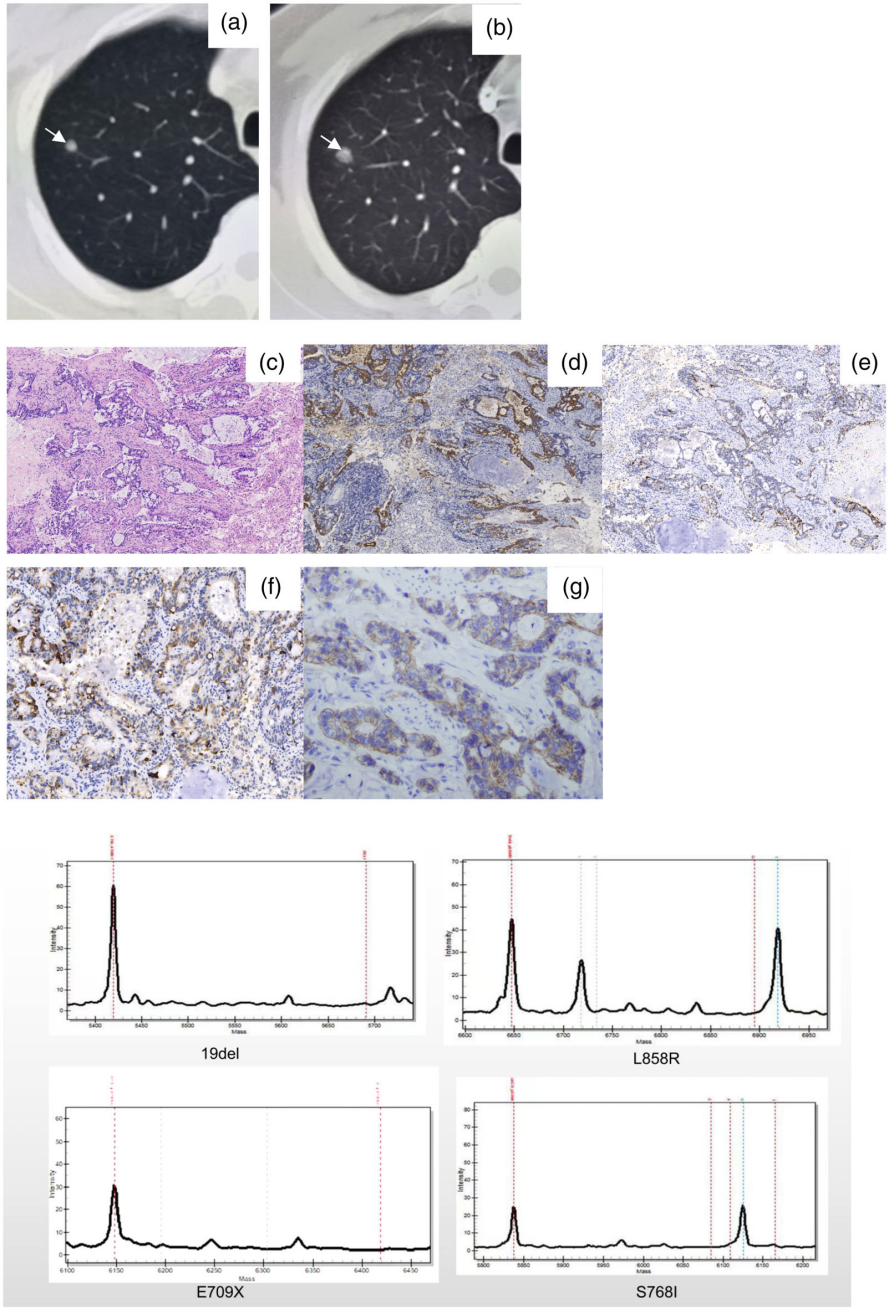
Chest CT scan detected single nodules in the right upper lung lobe 4 years post‐first surgery (a, white arrow), after 3 months, the size of the nodule increased (b, white arrows). (c) Microscopic appearance of the lung metastatic tumor showing invasive adenocarcinoma feature of irregular glands with mucin; (d) The metastatic lung tumor specimen exhibited positive expression for CK20; (e) The metastatic lung tumor specimen exhibited positive expression for CDX‐2; (f) The metastatic lung tumor specimen exhibited positive expression for MUC‐2; (g) The metastatic lung tumor specimen exhibited positive expression for β‐catenin (cytoplasmic, not nuclear); (d–g) Immunohistochemical staining, (d) The metastatic lung tumor specimen showed positive expression of CK20, (e) CDX‐2, (f) MUC‐2, (g) β‐catenin (cytoplasm, not nucleus). (g) Direct DNA sequencing of lung metastatic nodule specimens indicated that no mutation was found in 18–21 exons of the EGFR gene.

The postoperative course was uneventful, and the patient was followed without further treatment. A CT scan in December 2021 (4.25 years, post‐first surgery) revealed a 9‐mm shadow in the upper right lung lobe, which had grown during 3 months from 6 mm in September 2021 (post‐surgery 4 years) (Figure 2a,b). Video‐assisted thoracoscopic surgery (VATS) segmentectomy was performed, subsequently. Intraoperative and postoperative histological analysis confirmed mucinous adenocarcinoma with high similarity to the original tumor, and the margins were tumor‐free (Figure 2c). IHC showed positive expression of CK20, CDX‐2, MUC‐2, CK7, and negative β‐catenin (nuclear), GATA3, TTF‐1, Napsin A, and PD‐L1 (Figure 2d–g). Genetic testing of the lung specimen showed no mutation in EGFR (exon 18–21) (Figure 2h). The patient was diagnosed with Sheldon stage IVB urachal mucinous adenocarcinoma with lung metastasis.

## DISCUSSION

Urachal carcinoma was first described by Begg in 1931.^11^ Sheldon et al. (1984) proposed criteria for diagnosing urachal carcinoma,^7^ later refined by Gopalan et al. (2009).^12^ The Canadian Urological Association and Genitourinary Medical Oncologists of Canada (2020) further modified the criteria, which include (1) tumor location in the bladder dome or anterior wall, (2) tumor epicenter within the bladder wall, (3) absence of widespread adenomatous changes and cystitis cystica/glandular cystitis in the bladder wall, (4) absence of urothelial neoplasia, and (5) absence of a primary tumor elsewhere.^13^

While clinically frequently applied urine cytology may be negative for tumors located outside the bladder, multiple imaging techniques help in diagnosis. CT scans are essential in providing detailed information, and abdominal ultrasonography easily detects heterogeneous bladder wall masses (both hypo‐ and hyper‐echoic). Specifically, on CT, urachal carcinoma may appear as solid, cystic, or a mixed pattern, with low‐attenuation areas noted in 60% of cases due to mucin content; meanwhile, 50%–70% of cases presented calcification. MRI is also frequently employed to evaluate local invasion, nodal status, and distant metastases.

Additionally, diagnosing urachal carcinoma is challenging due to the histopathological feature overlap with adenocarcinomas arising from other organs, such as the appendix, pancreas, prostate, colorectum, biliary tract, and ovary. IHC plays a crucial role in differentiating urachal adenocarcinoma from other primary tumors. Urachal carcinomas generally exhibit positive expressions of CK7, CK20, and CDX2, with absent nuclear beta‐catenin expression. In contrast, prostatic and colorectal adenocarcinomas often involve the bladder neck or posterior wall rather than the dome. Prostate adenocarcinomas express prostate‐specific antigen and prostate‐specific acid phosphatase, while colorectal adenocarcinomas usually lack the high molecular weight cytokeratin 34 beta E12 and CK7 and show nuclear beta‐catenin expression.

In the present case, the IHC panel, combined with imaging findings of a bladder anterior wall tumor, strongly suggested urachal carcinoma over primary bladder cancer or adenocarcinomas from other organs. Additionally, the lung mass was histologically similar to the resected urachal carcinoma, with positive expression of CK7, CK20, Villin, and CDX2, and negative for GATA3, TTF1, and NapsinA, supporting the diagnosis of metastatic urachal carcinoma rather than primary lung adenocarcinoma.

Due to the rarity of urachal carcinoma, evidence‐based treatment guidelines are lacking. And urachal carcinoma is currently managed similarly to urothelial bladder carcinoma, with surgery being the primary treatment modality. The standard surgical approach involves excision of the urachus, umbilicus, and partial or radical cystectomy, often combined with bilateral pelvic lymphadenectomy. Notably, studies have shown no significant survival difference between partial and radical cystectomy.^14^, ^15^ Chemotherapy, typically cisplatin‐ and 5‐FU‐based, is recommended for patients with positive margins, local recurrence, or metastasis. Cisplatin‐based regimens combined with 5‐fluorouracil (5‐FU) have shown the lowest progression rates in patients. The protocol from MD Anderson Cancer Center recommended four to six chemotherapy cycles, depending on patient tolerance. Metastasis is most observed in the lung and regional lymph nodes. To date, only four cases of lung metastases from urachal carcinoma have been reported.^16^, ^17^ In all cases, the primary tumor and lung metastases were surgically removed, with lung metastasis occurring post‐surgery 4 months to 4 years. Radiotherapy is performed only in one case out of four, while adjuvant chemoradiotherapy, primarily involving 5‐FU and cisplatin‐based combinations, was administered to all four cases with most patients responding favorably. Reported survival time under chemotherapy ranged from 14 months to 11 years. Cases above suggested that a multimodal treatment strategy may offer the best chance for prolonged patient survival. Targeted therapies could also provide an alternative treatment approach, though more research is required to understand the molecular characteristics of urachal carcinoma and optimize treatment strategies.

Presently, systemic cancer treatments are shifting toward rationally individualized targeted therapies, especially for rare cancers including urachal carcinoma. Although data on the efficacy of targeted therapies in urachal carcinoma remain limited, there are some promising findings. Studies report that KRAS, NRAS, and BRAF mutations are present in 32%–57% of urinary duct cancer cases, and microsatellite instability (MSI) is present in 43% of cases. The expression of RAS/RAF/PI3K pathway alterations and PD‐L1 expression were detected in 16% of cases, suggesting a potential role for anti‐PD‐1/PD‐L1 immunotherapy and anti‐EGFR tyrosine kinase inhibitors (TKIs).^18^, ^19^

In the current case described, partial cystectomy and complete urachus, umbilicus, and bladder dome was performed. Pathologic examination revealed moderately differentiated mucinous adenocarcinoma without lymph node involvement, and surgical margins were negative. Four years postoperatively, a follow‐up CT scan detected a growing nodule (from 6 mm into 9 mm in 3 months) in the upper lobe of the right lung. Then the metastatic tumor was excised, with the surgical margins again negative. No chemotherapy was administered following either surgery. Prognostic factors in urachal carcinoma include surgical treatment, tumor differentiation, T‐stage, and negative surgical margins. In this case, the patient's good prognosis may be attributed to the well‐differentiated tumor, negative margins, and successful resection of lung metastases. Since genetic sequencing revealed no EGFR mutations (exon 18–21), targeted therapies were not pursued and the patient has shown no signs of recurrence so far.

## CONFLICT OF INTEREST STATEMENT

No author reports any conflicts of interest.

## Data Availability

The data are available from the corresponding author on reasonable request.

## References

[1] Szarvas T , Módos O , Niedworok C , Reis H , Szendröi A , Szász MA , et al. Clinical, prognostic, and therapeutic aspects of urachal carcinoma‐A comprehensive review with meta‐analysis of 1010 cases. Urol Oncol. 2016;34(9):388–398. 27267737 10.1016/j.urolonc.2016.04.012

[2] Tyler DE . Epithelium of intestinal type in the normal urachus, a new theory of vesical embryology. J Urol. 1964;92:505–507. 14226479 10.1016/S0022-5347(17)63995-8

[3] Peterson RM , Ollayos C , Merchant D . Urachal adenocarcinoma: incidental finding at the time of surgery for ruptured appendicitis. JSLS. 2006;10(3):392–395. 17212903 PMC3015710

[4] Wright JL , Porter MP , Li CI , Lange PH , Lin DW . Differences in survival among patients with urachal and nonurachal adenocarcinomas of the bladder. Cancer. 2006;107(4):721–728. 16826584 10.1002/cncr.22059

[5] Pinthus JH , Haddad R , Trachtenberg J , Holowaty E , Bowler J , Herzenberg AM , et al. Population based survival data on urachal tumors. J Urol. 2006;175(6):2042–2047. 16697798 10.1016/S0022-5347(06)00263-1

[6] Van Allen J . A rare case of urachal adenocarcinoma with bone marrow metastasis. BMJ Case Rep. 2021;14(4):e242315. 10.1136/bcr-2021-242315 PMC8054045 33858907

[7] Sheldon CA , Clayman RV , Gonzalez R , Williams RD , Fraley EE . Malignant urachal lesions. J Urol. 1984;131(1):1–8. 6361280 10.1016/s0022-5347(17)50167-6

[8] Ashley RA , Inman BA , Sebo TJ , Leibovich BC , Blute ML , Kwon ED , et al. Urachal carcinoma: clinicopathologic features and long‐term outcomes of an aggressive malignancy. Cancer. 2006;107(4):712–720. 16826585 10.1002/cncr.22060

[9] Reis H , van der Vos KE , Niedworok C , Herold T , Módos O , Szendrői A , et al. Pathogenic and targetable genetic alterations in 70 urachal adenocarcinomas. Int J Cancer. 2018;143(7):1764–1773. 29672836 10.1002/ijc.31547

[10] Collazo‐Lorduy A , Castillo‐Martin M , Wang L , Patel V , Iyer G , Jordan E , et al. Urachal carcinoma shares genomic alterations with colorectal carcinoma and may respond to epidermal growth factor inhibition. Eur Urol. 2016;70(5):771–775. 27178450 10.1016/j.eururo.2016.04.037 PMC5489411

[11] Begg RC . The urachus: its anatomy, histology and development. J Anat. 1930;64(Pt 2):170–183. 17104266 PMC1250190

[12] Gopalan A , Sharp DS , Fine SW , Tickoo SK , Herr HW , Reuter VE , et al. Urachal carcinoma: a clinicopathologic analysis of 24 cases with outcome correlation. Am J Surg Pathol. 2009;33(5):659–668. 19252435 10.1097/PAS.0b013e31819aa4ae PMC4225778

[13] Hamilou Z , North S , Canil C , Wood L , Hotte S , Sridhar SS , et al. Management of urachal cancer: a consensus statement by the Canadian Urological Association and Genitourinary Medical Oncologists of Canada. Can Urol Assoc J. 2020;14(3):E57–E64. 31348743 10.5489/cuaj.5946 PMC7053367

[14] Siefker‐Radtke AO , Gee J , Shen Y , Wen S , Daliani D , Millikan RE , et al. Multimodality management of urachal carcinoma: the M. D. Anderson Cancer Center experience. J Urol. 2003;169(4):1295–1298. 12629346 10.1097/01.ju.0000054646.49381.01

[15] Ashley RA , Inman BA , Sebo TJ , Fei X , Zong Y , Chen X , et al. Urachalcarcinoma:clinico‐ pathologic features and long‐term outcomes of an aggressive malignancy. Cancer. 2006;15(107):712–720. 10.1002/cncr.22060 16826585

[16] Kawakami S , Kageyama Y , Yonese J , Fukui I , Kitahara S , Arai G , et al. Successful treatment of metastatic adenocarcinoma of the urachus: report of 2 cases with more than 10‐year survival. Urology. 2001;58(3):462. 10.1016/s0090-4295(01)01259-6 11549502

[17] Somiya S , Aoyama A , Yamasaki T , Inoue T , Ogawa O , Kobayashi T . Successful surgical management of recurrent urachal adenocarcinoma: a case report. Urol Case Rep. 2020;32:101196. 32322529 10.1016/j.eucr.2020.101196 PMC7171456

[18] Kardos J , Wobker SE , Woods ME , Nielsen ME , Smith AB , Wallen EM , et al. Comprehensive molecular characterization of urachal adenocarcinoma reveals commonalities with colorectal cancer, including a hypermutable phenotype. JCO Precis Oncol. 2017;1:PO.17.00027. 32913973 10.1200/PO.17.00027 PMC7446420

[19] Módos O , Reis H , Niedworok C , Rübben H , Szendröi A , Szász MA , et al. Mutations of KRAS, NRAS, BRAF, EGFR, and PIK3CA genes in urachal carcinoma: Occurence and prognostic significance. Oncotarget. 2016;7(26):39293–39301. 27283768 10.18632/oncotarget.9828 PMC5129933

